# Analysis of drug–endogenous human metabolite similarities in terms of their maximum common substructures

**DOI:** 10.1186/s13321-017-0198-y

**Published:** 2017-03-09

**Authors:** Steve O’Hagan, Douglas B. Kell

**Affiliations:** 1 School of Chemistry, The University of Manchester, 131 Princess St, Manchester, M1 7DN UK; 2 Manchester Institute of Biotechnology, The University of Manchester, 131 Princess St, Manchester, M1 7DN UK; 3 Centre for the Synthetic Biology of Fine and Speciality Chemicals (SYNBIOCHEM), The University of Manchester, 131 Princess St, Manchester, M1 7DN UK

**Keywords:** Drug transporters, Cheminformatics, Endogenites, Metabolomics, Encodings, Maximum common substructure

## Abstract

In previous work, we have assessed the structural similarities between marketed drugs (‘drugs’) and endogenous natural human metabolites (‘metabolites’ or ‘endogenites’), using ‘fingerprint’ methods in common use, and the Tanimoto and Tversky similarity metrics, finding that the fingerprint encoding used had a dramatic effect on the apparent similarities observed. By contrast, the maximal common substructure (MCS), when the means of determining it is fixed, is a means of determining similarities that is largely independent of the fingerprints, and also has a clear chemical meaning. We here explored the utility of the MCS and metrics derived therefrom. In many cases, a shared scaffold helps cluster drugs and endogenites, and gives insight into enzymes (in particular transporters) that they both share. Tanimoto and Tversky similarities based on the MCS tend to be smaller than those based on the MACCS fingerprint-type encoding, though the converse is also true for a significant fraction of the comparisons. While no single molecular descriptor can account for these differences, a machine learning-based analysis of the nature of the differences (MACCS_Tanimoto vs MCS_Tversky) shows that they are indeed deterministic, although the features that are used in the model to account for this vary greatly with each individual drug. The extent of its utility and interpretability vary with the drug of interest, implying that while MCS is neither ‘better’ nor ‘worse’ for every drug–endogenite comparison, it is sufficiently different to be of value. The overall conclusion is thus that the use of the MCS provides an additional and valuable strategy for understanding the structural basis for similarities between synthetic, marketed drugs and natural intermediary metabolites.

**Electronic supplementary material:**

The online version of this article (doi:10.1186/s13321-017-0198-y) contains supplementary material, which is available to authorized users.

## Background

It is becoming increasingly clear that the transmembrane transport of drugs and xenobiotics via any trans-phospholipid bilayer diffusion is probably negligible, and thus that they have to “hitchhike” on the transporters of intermediary metabolism in order to get into cells [1–19]. Consequently, we [2, 20–22] and others (e.g. [23–27]) have recognised, on the basis of the principle of ‘molecular similarity’ [28–30], that successful, marketed drugs ought to bear structural similarities to endogenous (intermediary) metabolites (that we shall sometimes call ‘endogenites’ [2]).

Following an earlier sortie [2], we have used the availability of a carefully curated reconstruction of the human metabolic network, Recon2 [31–33], to answer this question in a straightforward manner. Now ‘similarity’, as an essentially ‘unsupervised’ concept, depends on the metrics of similarity used, and arguably is best judged post hoc simply in terms of its utility [29, 34]. Most strategies for assessing the similarities of small molecules use a means of encoding their 2D structures as bitstrings and comparing the similarities of those bitstrings (e.g. [29, 30, 35–41]). Thus, for the drug–endogenite comparison, it was clear that even using the common Jaccard/Tanimoto similarity metric the rank and magnitude of the similarities could vary widely between different encodings [20].

However, there are many different similarity coefficients even for (binary) bitstrings (Todeschini and colleagues compared 51 [42]), and just using the MACSS166 encoding [43] and the Tversky similarity [44, 45] with different α and β coefficients we again found an enormous variation (both qualitative and quantitative) [22] in the similarities determined between two molecules as α and β were varied. A particular recognition here, however, was the utility of interrogating with just sub-fractions of the molecule that were effectively exploited when α and β (at a constant α + β) were least similar to each other.

One scoring that is resistant to the detailed encoding used is based on the simple presence or absence of a given substructure, and assessing the frequencies and presence of some 600 common substructures provided a novel and useful metric, even with Tanimoto [21]. Again, however, the magnitude of the similarities determined depended on what fraction of the substructures (ranked in terms of their frequency) were used [21], and this encoding did not directly favour larger substructures over smaller ones.

All of these have been of value in recognising that approved, marketed drugs did share structural similarities with endogenous metabolites. A related question surrounds the “natural” substrates of particular transporters that transport pharmaceutical drugs, but this could not directly be answered from similarity considerations alone.

One structural feature that is largely (but not entirely, e.g. [46]) independent of both the encoding and the similarity used, at least if represented as a 2D graph of linked atom types, is the ‘maximum common substructure’ between two molecules (variously referred to as the MCS or MCSS). It has achieved especial prominence because of the frequent use of ‘scaffolds’ in medicinal chemistry, where the scaffold is effectively equivalent to a large, common substructure (e.g. [47–52]). Although its calculation is computationally much more demanding than are many of the other calculations in similarity cheminformatics [46, 53–65] (and see below), this essential independence from both the encoding and the similarity metric means that it is a principled strategy that we considered worth exploring for the drug–metabolite similarity problem. It was not necessarily clear that MCS would be better, but it was recognised that it would provide different information; in particular an MCS is a graph of connected atoms, with a clear chemical meaning, while a fingerprint is essentially uninterpretable without knowledge of how it was generated (and in many cases, e.g. for isomers, it is not unique, whereas an MCS is an MCS). The results of this analysis are given here.

## Methods

The list of endogenous metabolites and marketed drugs was precisely as used previously [20–22], and we used the KNIME workflow environment (e.g. [66–72]) to write the appropriate workflows for these analyses. In particular, we used the RDKit [73] (http://rdkit.org/) MCS nodes for the MCS calculations. To provide a metric for the MCS, we followed the recent analyses of Bajorath and colleagues [65, 74, 75]. Thus they recognised that a similarity equivalent to the Tanimoto similarity for a molecule A with a total of |A|_b_ heavy atoms and another molecule B with |B|_b_ heavy atoms, could be written in the form [74]

1 $$ {\text{Tc}}_{\text{MCS}} (A,B) = \frac{{\left| {{\text{MCS}}(A,B)} \right|_{b} }}{{|A|_{b} + |B|_{b} - |{\text{MCS}}(A,B)|}}_{b} $$

where |MCS(A, B)|_b_ is the number of heavy atoms in the MCS. Elementary inspection of Eq. (1) shows that the value of the Tc_MCS_ does, as expected, range between 0 and 1.

The Tversky similarity coefficient Tv(A, B) coefficient [44, 76–78] is defined as:

2 $$ {\text{Tv}}\left( {{\text{A}},{\text{B}}} \right) = {\text{c}}/(\upalpha{\text{a}} +\upbeta{\text{b}} + {\text{c}}), $$

where a and b are the number of bits that are set to be ‘on’ (1 bits) only in molecular fingerprints A or B, respectively, and c is the number of on bits shared by both A and B. A is an interrogatory molecule while B is the molecule being interrogated as to its similarity. The smaller the value of α, the larger the contribution of B as a substructure of A (and hence to its similarity with A). The larger the value of α, the larger the contribution of B as a superstructure of A (equivalently A as a substructure of B). For α = β = 1 the coefficient is numerically equivalent to the Tanimoto similarity.

A similar strategy could be followed [65, 75] (Eq. 3) to report a Tversky similarity as per Eq. 2, with α and β having their usual meanings as in the previous paragraph [22, 44, 76–78]. As before, we studied the effect of varying α while the sum of α and β was either 1 or 2.

3 $$ {\text{Tv}}_{\text{MCS}} (A,B,\alpha ,\beta ) = \frac{{|{\text{MCS}}(A,B)|_{b} }}{{\alpha (|A|_{b} - |{\text{MCS}}(A,B)|_{b} ) + \beta (|B|_{b} - |{\text{MCS}}(A,B)|_{b} ) + |{\text{MCS}}(A,B)|_{b} }},\quad \alpha ,\beta \ge 0 $$

Specifically, the MCS algorithm used in this study was the fast connected MCS algorithm fMCS, as implemented in RDKit (see http://www.dalkescientific.com/writings/diary/archive/2012/05/13/fmcs.html and http://rdkit.org/Python_Docs/rdkit.Chem.fmcs.fmcs%27-pysrc.html). We used Python 2.7 + the Python RDKit package to generate [for all A and B’s] the MCS SMARTS string, the a,b, and MCS Atom counts; as well as the Tanimoto-like MCSS.

## Results

### One drug versus all drugs plus endogenites

**Fig. 1:**
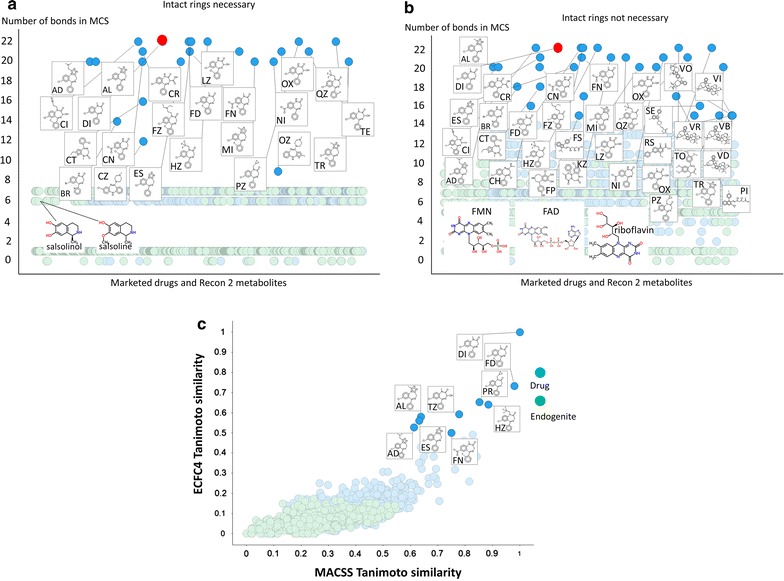
Maximal common substructure (MCS) between diazepam (in *red*) and other marketed drugs and metabolites. The size of the MCS is plotted for various drugs (*blue*) and endogenous metabolites (*green*). A KNIME workflow was constructed, including using the RDKit MCS module and interrogated with the structure of diazepam. **a** Distribution of MCS values when the RDKit MCS was set to use only intact rings. **b** The same without that restriction. In both cases, the structures of the closest molecules are shown. **c** A comparison of the Tanimoto similarity of diazepam and other drugs and endogenites using two common fingerprint encodings (ECFP4 and MACCS). The structures of those exceeding 0.5 in each encoding are shown.

**Fig. 2:**
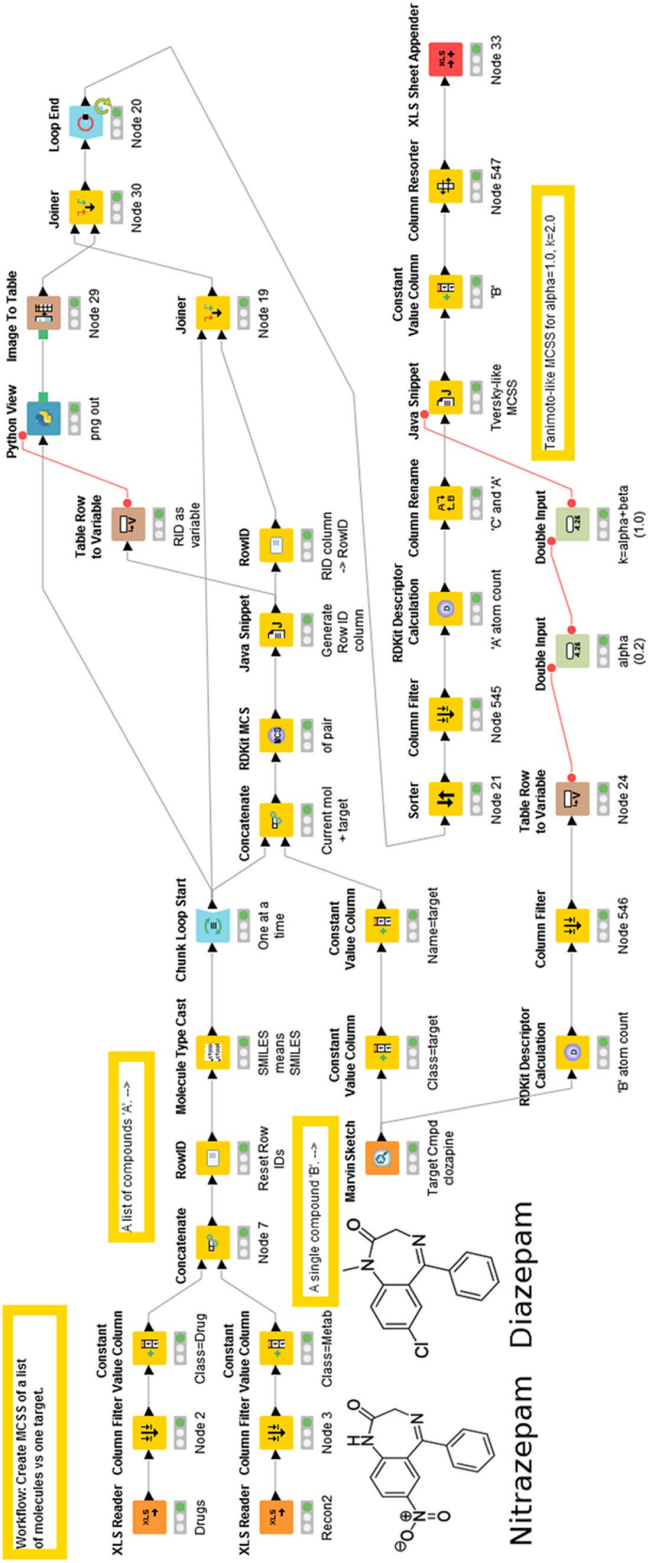
The KNIME workflow was used to construct Fig. 1 (also included as Additional file 1), including using the RDKit MCS module, and interrogated with the structure of diazepam. The Python code used (see “Methods”) is given as Additional File 2

In our previous work [20], where we clustered marketed drugs on the basis of their chemical structures, this was simply a prelude to comparing them with metabolites but we did not dig down into the clusters so formed at any level of detail. Here, it was of initial interest to establish whether the MCS strategy did indeed return as most similar drugs containing a particular scaffold. To this end, we chose diazepam, as an example of a ‘first generation’ antipsychotic. As expected, it showed a shared pedigree with other related benzodiazepine molecules (Fig. 1). Such molecules were less similar to ‘second generation’ molecules such as clozapine and olanzapine [79–81] that are themselves part of a (large) family of such molecules with a complex pharmacological profile [82]. Figure 1a shows the various molecules as a function of the number of heavy atoms in the MCS when whole (aromatic) rings must be present in the MCS. Only 23 molecules have 9 or more heavy atoms in the MCS (Fig. 1a). All are well known antipsychotic drugs. The metabolites with the largest MCS (6 heavy atoms) are salsoline and salsolinol (which is not unreasonable, as they are condensation products of dopamine and acetaldehyde [83–87]). When this ‘whole-ring’ assumption is relaxed (Fig. 1b), a somewhat different pattern emerges, though we mark only those molecules with at least 16 heavy atoms in the MCS. Now the closest three metabolites (FAD, FMN and riboflavin) have 11 heavy atoms in the MCS, and while this strategy retains the main molecules of the ‘rings-only’ strategy, it now lets in molecules such as ‘statins’ (fluvastatin, pitastatin), anticancer *Vinca* alkaloids (vinblastine, vincristine, vindesine), and quinolone antibiotics (rosoxacin) whose basic scaffold is really nothing like that of a benzodiazepine. Note that Fig. 1 consists in total of 1112 metabolites and 1381 marketed drugs, making 2493 marketed drugs plus endogenous metabolites in toto. All 23 diazepams cluster together, and their lowest TS to diazepam when the encoding is the MCS is 0.667. By contrast, many more substances appear similar when some of the classical fingerprints are used. Figure 1c shows the Tanimoto similarities for diazepam versus all drugs (blue) and endogenites (green) for two RDKit encodings (MACCS and ECFP4), where 175 molecules have a MACCS-TS > 0.5, though only 9 molecules show similarities above 0.5 for both encodings. (The closest metabolites, which also do, are methylene tetrahydrofolate and vitamin D_2_.) The simplest interpretation is really that the MCS is much more discriminating for what it says, i.e. the maximum common substructure or scaffold, but that this leads to a more natural and useful clustering. Finally, here, Fig. 2 and Additional file 1 shows the workflow used for Fig. 1a, b, and illustrates how we indicated the MCS in the Excel sheet to which the analyses were output. Thus we preferred the MCS that required that if rings were present they had to be present in their entirety in both molecules to contribute to the MCS.

### MCS of all drugs and/or metabolites against each other

**Fig. 3:**
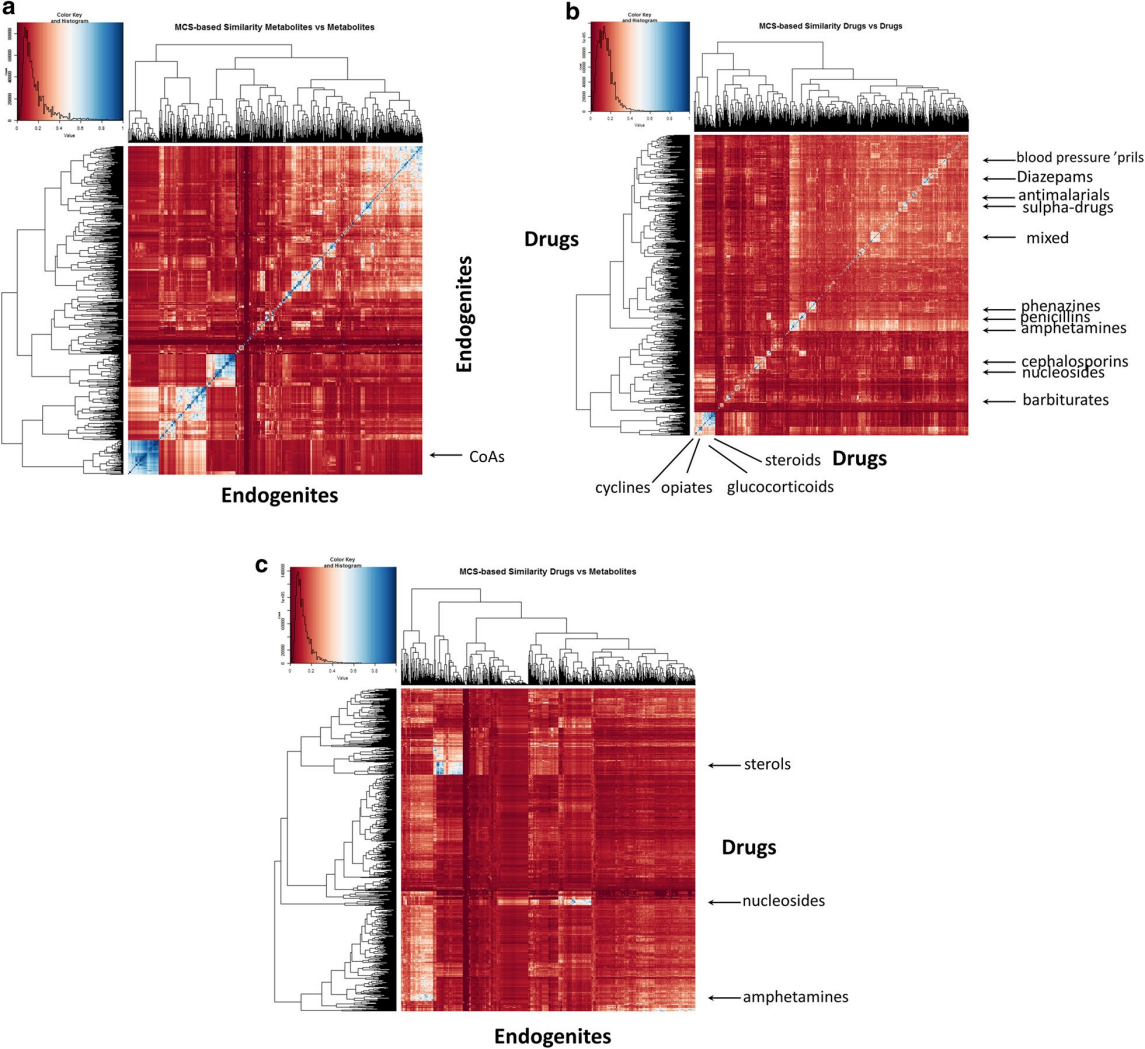
Heat map of the comparison of the Tanimoto similarities of the MCS for **a** endogenites versus endogenites, **b** drugs versus drugs, **c** drugs versus endogenites

While this was considerably more demanding in computer time than our previous similarity analyses based on various fingerprints coupled to Tanimoto or Tversky similarity [20–22, 88], it proved possible and useful to do. A run of all drugs against all metabolites took approximately 3 days on a reasonably modern PC (Intel i7-4930K, 6 cores hyperthreaded cpu (12 virtual cores) @ 3.4 GHz, 64 GB Ram). We here used MACCS166 as the ‘main’ fingerprint. Others such as ECFP (and FCFP etc.) were not done since (1) comparison of MCS versus all possible fingerprints would have been completely unwieldy, and (2) we had compared the fingerprints with each other in our previous papers. Since MACCS gave among the largest similarities [20], we also considered that it would provide the sternest ‘test’ of the utility of MCS. Figure 3 shows heat maps for the three comparisons (endogenites–endogenites, drugs–drugs, drugs–endogenites), analogous to those performed [20] using molecular encodings. Relevant Excel sheets are given in the Additional files 3, 4, 5 to allow readers to explore further, but these are very rich in information. Thus, although (Fig. 1a) they tend to give more ‘sensible’ hits where scaffolds exist, numerically they only attain large Tanimoto similarities for rather similar drug or endogenite classes. These classes may be seen as blue clusters in Fig. 3, some of which are marked therein. As before, there are larger endogenite clusters, where CoA derivatives (bottom left of Fig. 3a) and sterols (bluest cluster nearer the middle) again clearly dominate, in contrast to the much ‘bittier’ population of drug space (Fig. 3b). The largest clusters of similarity of drugs versus endogenites (Fig. 3c) are again sterols (largest blue cluster, towards the top left), with others (marked in Fig. 3c) including amphetamines (similar to various neurotransmitters such as (nor)adrenaline), and nucleosides.

**Fig. 4:**
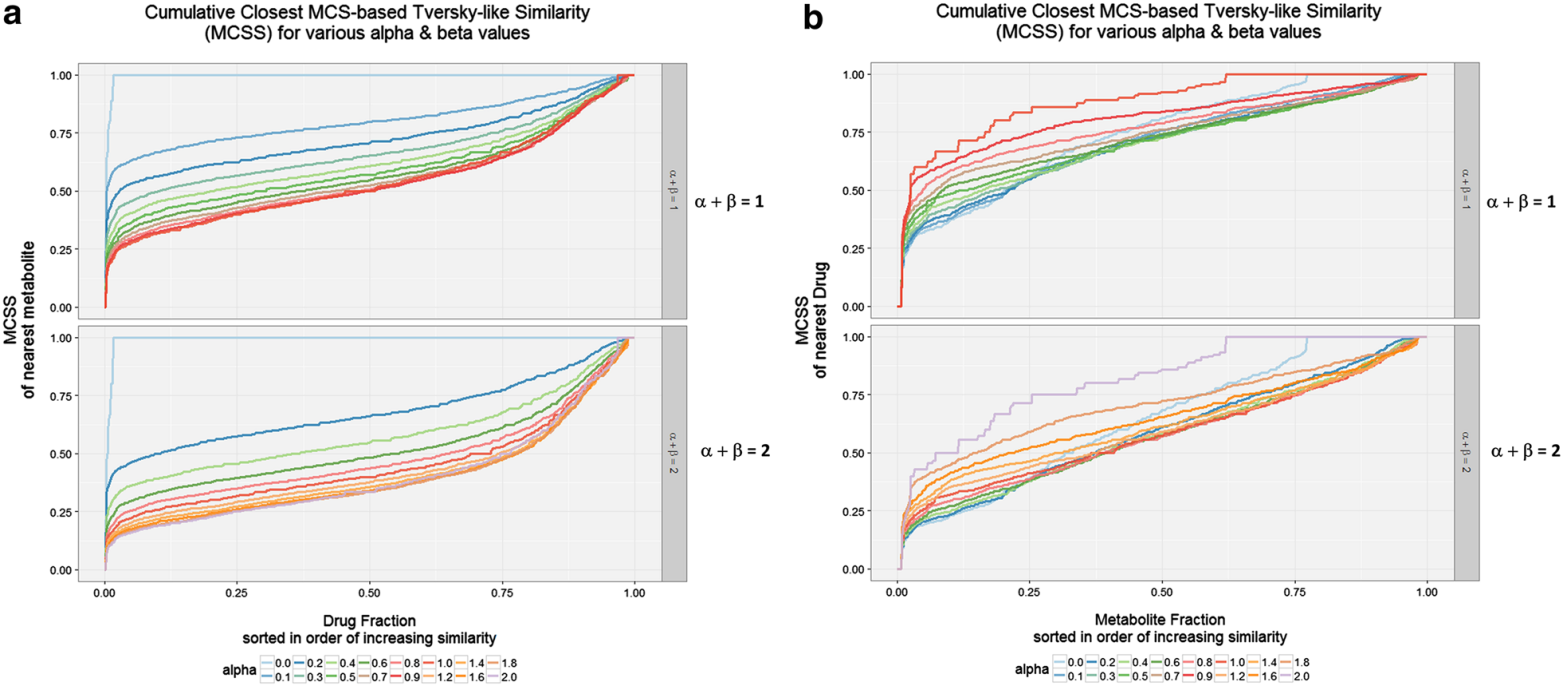
Cumulative Tversky similarities for various values of Tversky α and β of **a** a drug to its closest endogenite, **b** an endogenite to its closest drug

While the calculation of the MCS values was quite demanding, the calculation of other similarities (see “Methods” section) was much simpler, as those used depended only on the number of heavy atoms in the molecules being compared and those in their MCS. Since the Tversky similarity metric had proven (at some values of α and β) to be much more appropriate than Tanimoto for highlighting drug–endogenite similarities, we again used it. Comparing drugs (interrogating molecule) versus endogenites (interrogated library) it is clear (Fig. 4a) that for values of α such as 0.2 (when α + β = 1) the Tversky similarity of at least one endogenite for virtually every drug exceeds 0.5 when using the MCS as the encoding, whereas this is much less true from when the Tanimoto similarity (α = β = 1) is used (Fig. 4a). The same is true for the converse [where the interrogating molecule is an endogenite (Fig. 4b)].

### Some examples

It seems that the MCS method of molecular comparison, when all rings are included intact, gives much more reliable measurements of useful similarity as judged by scaffolds. As ever, the different metrics give different indications of how similar two molecules seem to be. To this end, we interrogated the endogenites with a few drugs carefully chosen to illustrate the kinds of variation observable, first illustrating their differences with (1) an MCS-based similarity with Tversky α 0.2 and β 0.8 and (2) a MACCS encoding and a Tanimoto similarity as in [20].

**Fig. 5:**
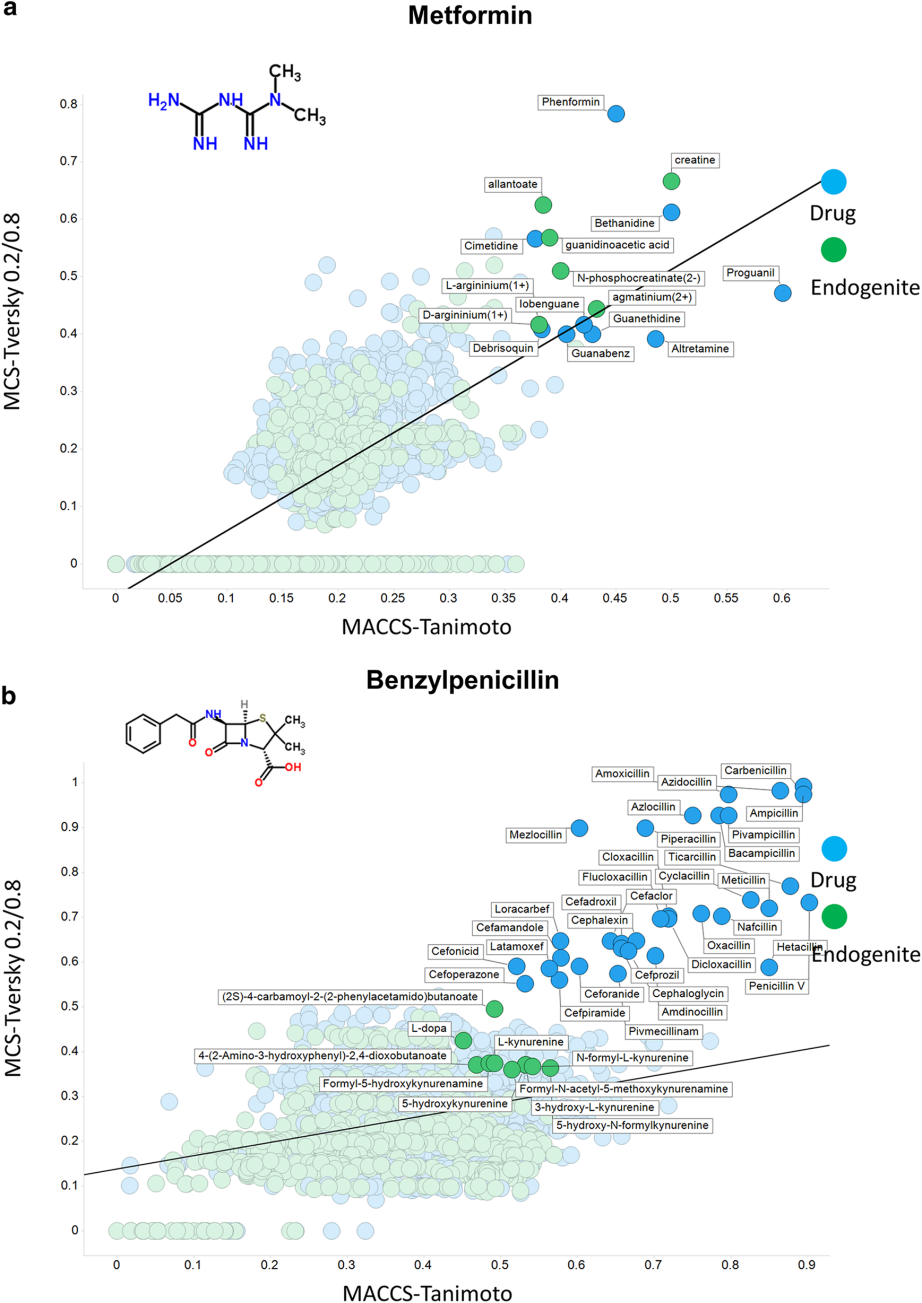
Relationship between MCS encoded as a Tversky similarity (α, β = 0.2, 0.8) and MACCS-encoded Tanimoto similarity from selected drugs with other marketed drugs (*blue*) and endogenous metabolites (*green*), highlighted at an arbitrary ‘break’ for each class and where the numbers involved were small enough to permit legibility. The *straight lines* are those of best fit. **a** Metformin. **b** Benzylpenicillin

**Fig. 6:**
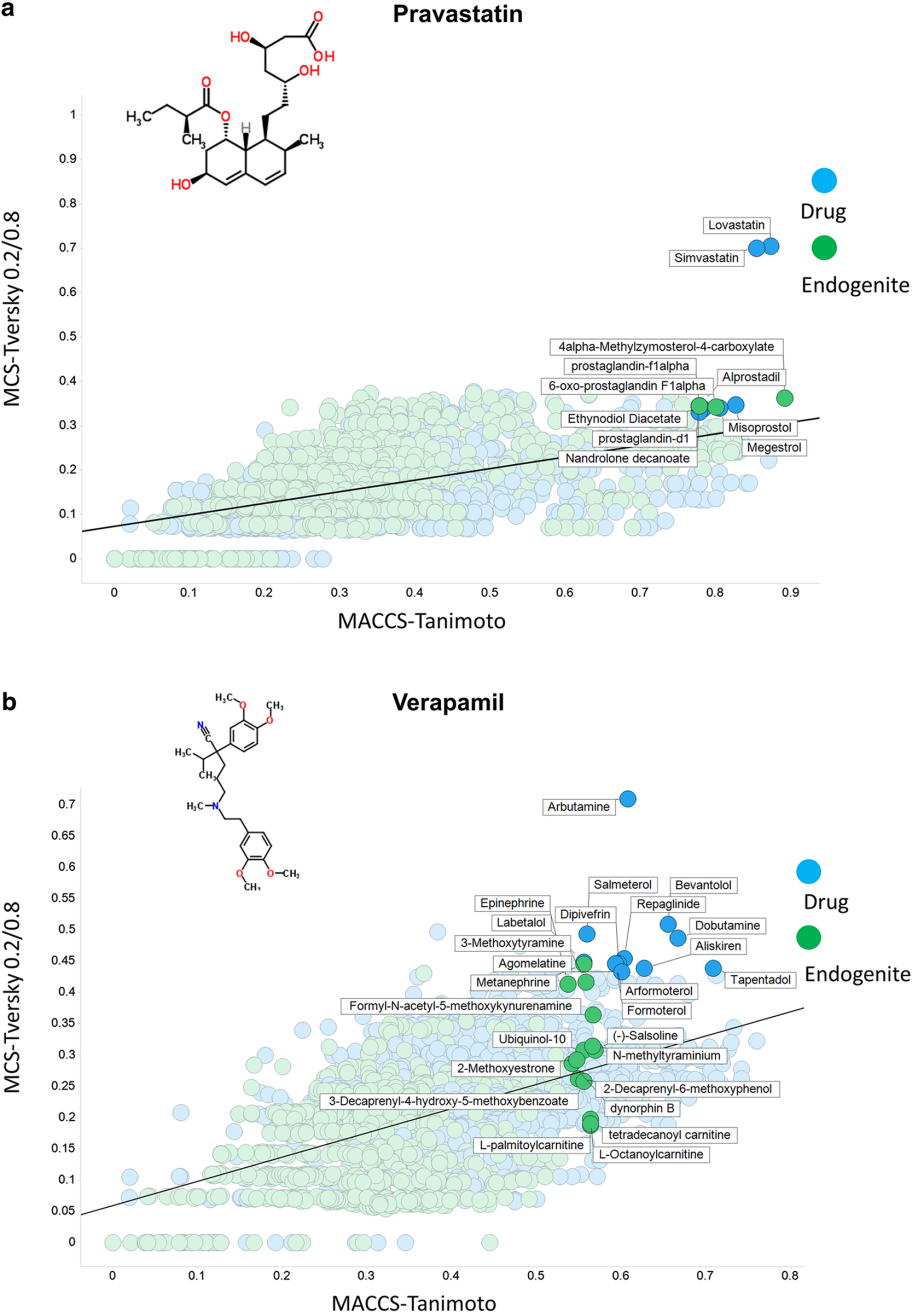
Relationship between MCS encoded as a Tversky similarity (α, β = 0.2, 0.8) and MACCS-encoded Tanimoto similarity from selected drugs with other marketed drugs (*blue*) and endogenous metabolites (*green*), highlighted at an arbitrary ‘break’ for each class and where the numbers involved were small enough to permit legibility. The *straight lines* are those of best fit. **a** Pravastatin. **b** Verapamil

Figure 5a shows the very small and hydrophilic metformin (MW 129.17), and how the MCS/Tversky encoding shows it to be much more metabolite-like than does the MACCS_Tanimoto analysis. Partly this is because its small size means that many bits are set low and so the TS is low (see [22, 89–91]). Nevertheless, its structural similarity to creatine (most similar via the Tversky metric) and other organic cations is consistent with the fact that it is taken up by SLC22 family members (known as Organic Cation Transporters in the older literature [92–99]). Benzylpenicillin (334.39) illustrates a couple of interesting features (Fig. 5b). First is that among the drugs (in blue) it clusters most closely with the penicillins and then with the cepahlosporins, as expected. Secondly, the metabolites to which it is most similar include several N-substituted kynurenine derivatives, consistent with an anticipation that at least some of them might share a similar transporter. This is in fact the case (SLC15 family, e.g. [100–104]). Pravastatin (MW 424.53) is one of the so-called ‘statin’ class of drug that can inhibit HMGCoA reductase. As is clear from Fig. 6a, apart from the related natural products simvastatin and lovastatin, it does not show any obvious similarity or major MCS to any other so-called statin (e.g. atorvastatin (Lipitor) or rosuvatstain (Crestor)), even though they all share a glutarate or related lactone group. Arguably this reflects the fact that much of their activity is in fact due to interactions (of the other parts of the molecule) with other targets (e.g. [105–119]), and expression profiling demonstrates clearly [120] that they lack a unitary mode of action. Consequently it is less surprising that MCS performs poorly in this regard, since they really do not have much of a common substructure. Verapamil (MW 454.6) is a Ca^++^-channel blocker with multiple disease indications (implying considerable promiscuity, consistent with a log P value of 3.79 http://www.drugbank.ca/drugs/DB00661). It is also considered one of the more rapidly transported drugs in Caco-2 cells (e.g. [14, 15]). According to ChEMBL https://www.ebi.ac.uk/chembldb/index.php/compound/inspect/CHEMBL6966, it interacts with some 172 targets, including 11 uptake transporters, which presumably accounts for this. The central core, consisting of a long, branched and predominantly carbon-based linker, and the heterogeneous nature of the molecules to which it is ‘similar’ (Fig. 6b), would also be consistent with this.

**Fig. 7:**
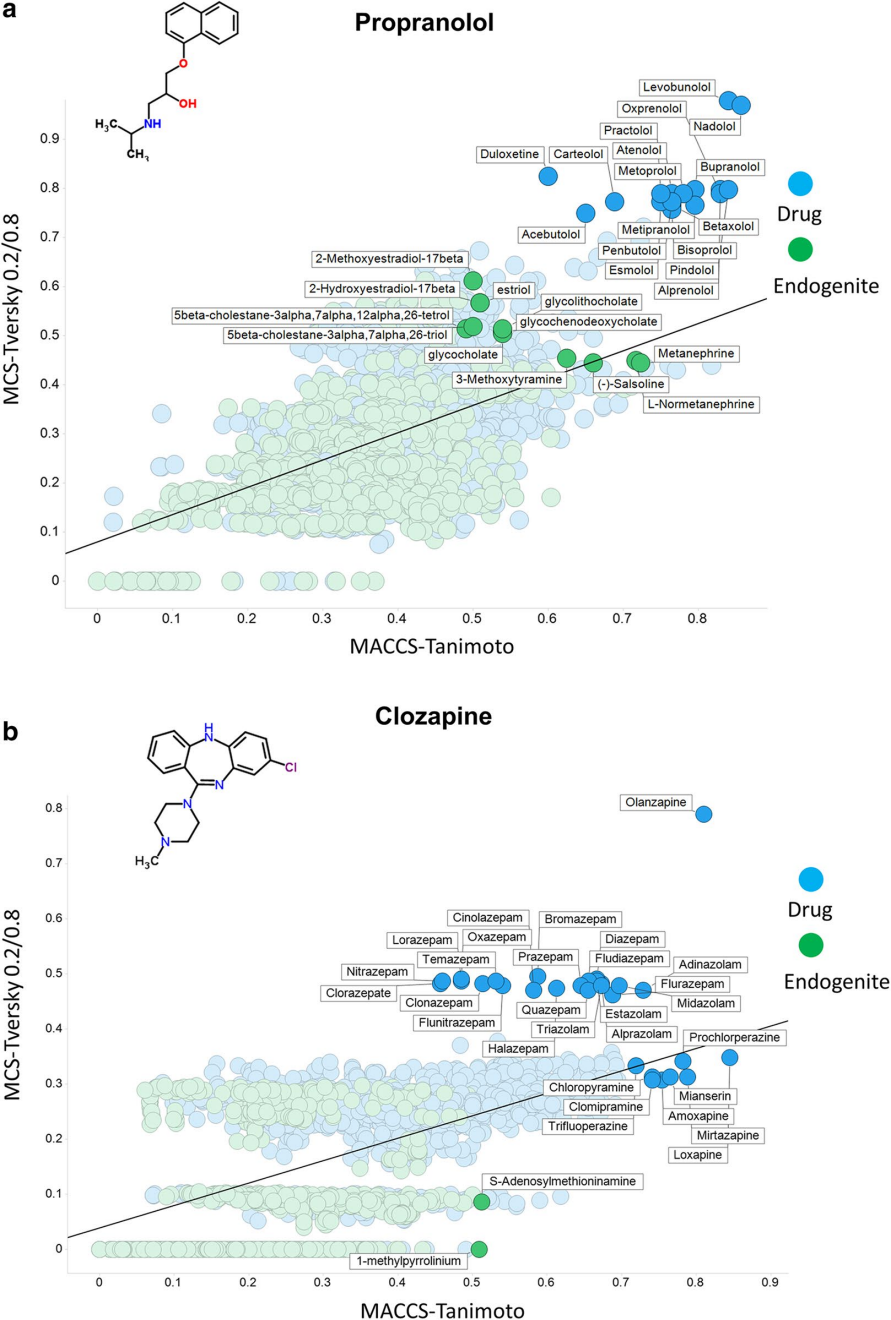
Relationship between MCS encoded as a Tversky similarity (α, β = 0.2, 0.8) and MACCS-encoded Tanimoto similarity from selected drugs with other marketed drugs (*blue*) and endogenous metabolites (*green*), highlighted at an arbitrary ‘break’ for each class and where the numbers involved were small enough to permit legibility. The *straight lines* are those of best fit. **a** Propranolol. **b** Clozapine

Propranolol (Fig. 7a) (MW 259.15), another drug enjoying a high rate of transport through Caco-2 cells [14, 15], is a classical β-adrenergic receptor blocker. Unsurprisingly, the analysis pulls out many analogues both as drugs and (for metabolites) among analogues of (nor)adrenaline (synonym (nor)epinephrine) such as metanephine. As judged by the data deposited in ChEMBL https://www.ebi.ac.uk/chembldb/index.php/compound/inspect/CHEMBL27 it has 166 known targets, including 9 uptake transporters. Its structural similarity to noradrenaline means that unsurprisingly these include the very active serotonin, dopamine and noradrenaline transporters. Finally, we show a drug that is among the least obviously metabolite-like, viz. clozapine (Fig. 7b), and also rather hydrophobic; only two endogenites have a Tanimoto similarity exceeding 0.5, though its similarity to related drugs is indeed reasonably high. (The same phenomena attach to sepantronium bromide, a potent drug candidate for which significantly more than 99% of uptake flux into cells occurs via a single transporter (SLC35F2) [11], and for which any phospholipid bilayer transport is consequently negligible [10, 13, 17, 121]; data not shown.)

Although the data are implicit in Figs. 5, 6, 7, it is worthwhile (Table 1) just tabulating the number of molecules for which the difference in the encodings (MACCS_TS–MCS_Tv) is positive and negative for the six molecules, as this makes it clear how much they can differ in either direction.

**Table 1:** Variation in sign

Molecule	Positive difference MACCS_TS–MCS_Tv	Negative difference MACCS_TS–MCS_Tv	% with a positive difference
Clozapine	1366	379	78.3
Metformin	1034	711	59.3
Benzylpenicillin	1282	463	73.5
Pravastatin	1575	170	90.3
Propranolol	1172	573	67.2
Verapamil	1496	249	85.7

### Accounting for differences in the similarity metrics

Even just with these six drug molecules, it is clear that the degree of similarity with endogenites varies both qualitatively and quantitatively depending on what is the drug and what is the encoding and similarity metric. To this end, we have determined the differences in the similarity between these drugs and endogenites for each endogenite, and sought to understand what in structural or descriptor terms might account for it (in the way that we know that low numbers of bits in the bitstring, as occurs more for smaller molecules, necessarily makes the MACCS Tanimoto similarity appear smaller [21, 36, 77, 89, 122–126]). To this end, we set up the following strategy:

Read Drugs + Recon2—the ‘A’ molecules. Then select the six named ‘B’ molecules, as in Figs. 5, 6, 7 and Table 1. Loop over each ‘B’. For each ‘A’ paired with a ‘B’ calculate the MACCS-TS & Tversky-like MCS (alpha = 0.2, beta = 0.8), and their difference Delta. Calculate all available scalar (non-vector) RDKit descriptors of each ‘A’—these are the input features of the model. Remove any constant features (there were none). Remove one of each pair of correlated features (r ≥ 0.98); 13 feature columns removed. Split into 70:30 train:test set. Use a Random Forest regression model (200 trees; see [127, 128]) to predict delta as the objective function. Collect the Out-of-box and Test predictions for each molecule ‘B’. Plot a Scatter plot of Actual versus Predicted for each ‘B’ on the test predictions [127].

**Fig. 8:**
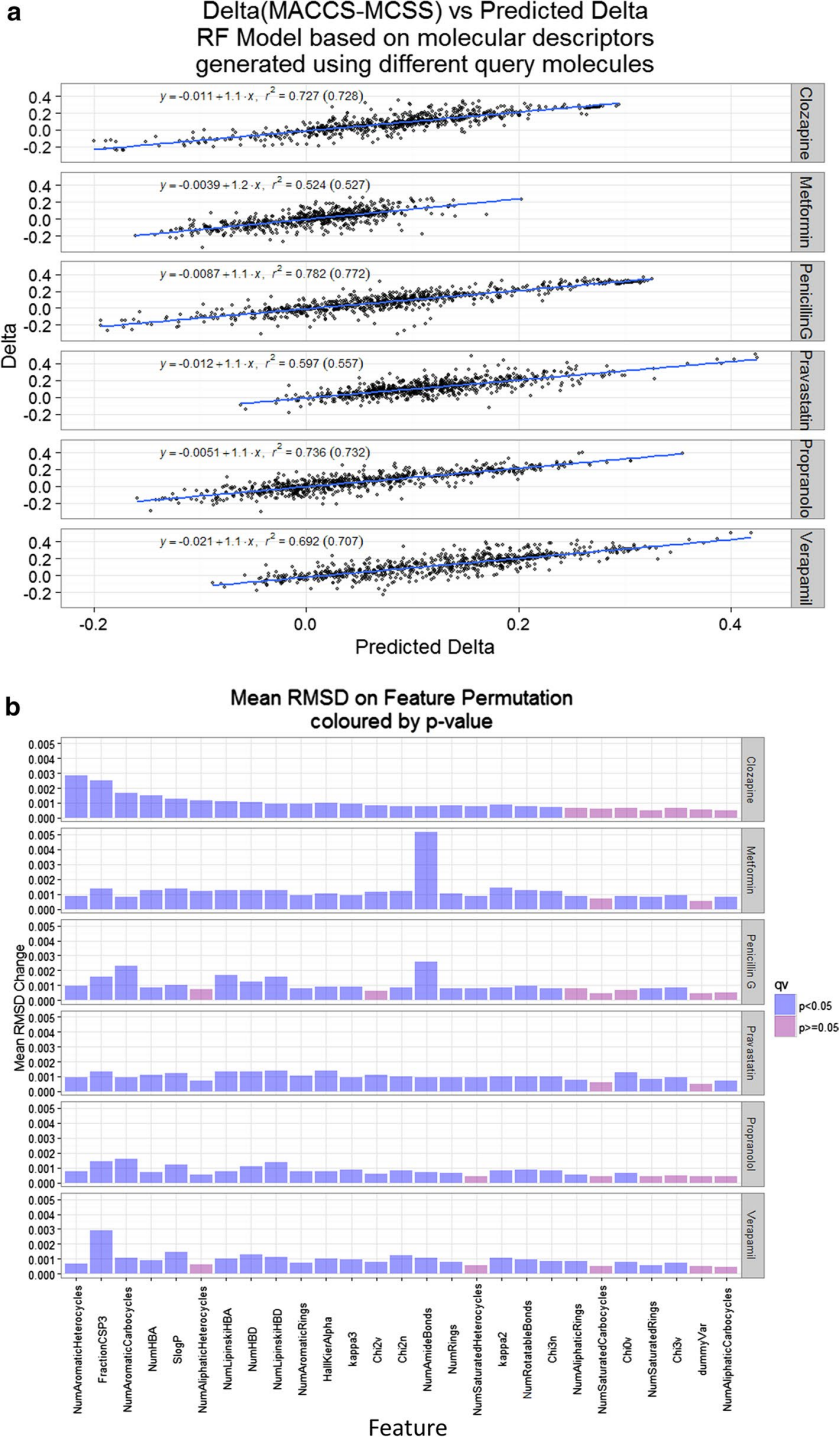
Random Forest prediction of the differences (Delta) between MACCS_Tanimoto and MCS_Tversky similarities. **a** *Scatterplot* with regression coefficients for 6 drugs. **b** Contribution of each of the retained RDKit features for each drug

Although trends varied for each of the 6 drugs in Figs. 5, 6, 7, no individual descriptor such as S log P could, on its own, account for the differences between MACCS_Tanimoto and MCS_Tversky. However, a random forest model could do so when out-of-bag tests were done, with the predictions and contributions of the descriptors given for the six drugs in Fig. 8. It is clear (1) that the differences are deterministic (Fig. 8a), but (2) that the basis for them, i.e. the features that contribute to those differences, is bespoke to each drug (Fig. 8b). The same was true of 10 other drugs selected at random (data not shown).

## Discussion

It is clear that, even when using MCS and Tversky similarities where most drugs do manifest a reasonable similarity to at least one endogenite, the closeness of that similarity can be quite variable. If the effectiveness of drugs is indeed related to their ability to interact with binding sites of proteins, including transporters, that also interact with natural metabolites, this bears some explanation. One straightforward explanation, of course, is simply that we still have to discover many of the naturally occurring metabolites, and that the excellent Recon2—based on metabolic enzymes that are encoded by the genome sequence plus a few vitamins—is useful only insofar as it knows about them. Several general kinds of argument imply that this may indeed be the case. The first is that we can detect many more small molecules as mass spectral signals in biological samples than we can presently identify [129], possibly as a result of unknown enzyme promiscuity [130–132]. Similarly, from the point of view of metabolic network reconstructions, the latest version of Recon2, Recon2.2 [33], contains 2652 unique chemical species, some 60% more than in Recon1 [31, 133], implying that we are far from discovering them all, and some are known still to be absent [9]. Thirdly, many of the metabolites may not be entirely the result of the host’s biosynthesis, being derived from dietary sources [134, 135] and including biotransformations in the gut. At an elementary level this is clearly true, since essential amino acids, fatty acids and vitamins are (by definition) not synthesised by the host. However, as known elements of human metabolism, these are generally taken into account and appear in the metabolic reconstructions, albeit many ‘known’ metabolites still do not [9]. The ability to transport such compounds may be of relatively recent evolutionary origin, much as is the ability of mammals to digest lactose in adulthood [136–138] (which is also highly variable between individuals and indeed races [139, 140]). We also note that the experimental serum metabolome listed at http://www.serummetabolome.ca/statistics [141] refers to 2243 endogenous metabolites but 3363 exogenous metabolites, with the corresponding numbers for the human urine metabolome [142] being 1665 endogenous metabolites and 3363 exogenous metabolites.

At all events, when we compared the differences in the magnitude of the similarity between MACCS_Tanimoto and MCS_Tversky, it was clear that they could be positive or negative, although MACCS was more often the larger, but that no individual descriptor could account for these differences, even though they were clearly deterministic (as are the analyses). Overall, though, it is clear that the use of the MCS adds significantly to the armoury of similarity strategies for those seeking to compare the structural similarities between synthetic drugs and natural biomolecules.

## Conclusion

The extent to which two molecules are to be seen as ‘similar’ in purely (2D) structural terms depends strongly on both their encoding and the similarity metric used, and this was the case for our drug–endogenite analyses as performed previously [20–22]. In the absence of ‘activity’ or ‘functional’ data, the only comparators for ‘closeness’ rely on purely unsupervised methods of analysis. It is clear that not all of a drug will typically bind to its ‘target’ (not least since some molecular features will have been designed in for other purposes, e.g. ADME). However, the extent of this is normally not known, and probably not knowable, and that necessarily underpins part of the functional variation in similarity.

One strategy to ensure that we pick up pertinent similarities is to use as many methods as possible for encoding them, and we here sought to assess the maximal common substructure (MCS) as an additional useful similarity measure. MCS also has the advantage of having a clear chemical meaning in terms of a linked set of atoms. Although, again, the extent to which the MCS showed up similarities observable via the MACCS fingerprint varied significantly between drugs, the corresponding conclusion was precisely that, as a consequence of this, the MCS was valuable as an additional method in such comparisons. To reiterate, we do not imply that MCS is ‘better’ or ‘worse’ than other methods, but we do think that the evidence shows that it is different and correspondingly valuable, and should thus be used in parallel with fingerprinting methods, whether separately or (as often done to advantage, e.g. [63, 143, 144]), via fusion methods. Finally, a referee wondered whether there might be a correlation between MCS-similarity to the nearest endogenite and bioavailability. The present analysis now opens up the possibility of answering precisely these and other such questions.

## Additional files

**Additional file 1.** Workflow of Fig. 2 used to generate the data shown in Fig. 1.

**Additional file 2.** Python code used to generate substructures.

**Additional file 3.** Comparison of endogenites with endogenites in terms of their maximum common substructures.

**Additional file 4.** Comparison of marketed drugs with marketed drugs in terms of their maximum common substructures.

**Additional file 5.** Comparison of endogenites with marketed drugs in terms of their maximum common substructures.
