# Supplementary figures and images for: Analysis of drug–endogenous human metabolite similarities in terms of their maximum common substructures
Source: J Cheminform. 2017 Mar 9;9:18. doi: 10.1186/s13321-017-0198-y (PMC5344883; doi:10.1186/s13321-017-0198-y)

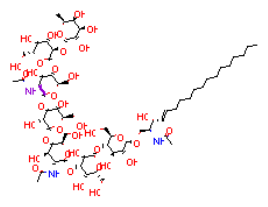

Supplement: Supplementary file 1 — Additional file 1. Workflow of Fig. 2 used to generate the data shown in Fig. 1. [file 13321_2017_198_MOESM1_ESM.zip › DM_MCS_Hits clozapine/Python View (#26)/internal/image.png]
